# Metabolizable Energy, Standardized Ileal Digestible Amino Acids, Standardized Total Tract Digestible Phosphorus, and Replacement Value of Full-Fat Almonds for Pigs

**DOI:** 10.3390/ani16193043

**Published:** 2026-09-27

**Authors:** Yujeong Hwang, Jong Woong Kim, Beob Gyun Kim

**Affiliations:** 1 Department of Animal Science, Konkuk University, Seoul 05029, Republic of Korea; g8661206@konkuk.ac.kr; 2 Easy Holdings Co., Ltd., Seoul 06253, Republic of Korea; eskimjw@easyholdings.co.kr

**Keywords:** almonds, amino acids, energy, phosphorus, pigs, replacement coefficients

## Abstract

This work aimed to determine the energy values and digestibility of amino acids (AA) in full-fat almonds, to estimate phosphorus (P) digestibility in full-fat almonds, and to calculate the replacement value of full-fat almonds in pig diets. In experiment 1, the digestible energy (DE) and metabolizable energy (ME) of full-fat almonds were determined using six barrows (34.5 ± 2.0 kg) housed in individual metabolism cages. A basal diet containing corn, soybean meal, and soybean oil was used, and the almond diet was formulated by replacing the energy sources in the basal diet with full-fat almonds at 10%. In experiment 2, AA digestibility was determined using six barrows (28.9 ± 1.0 kg) fitted with a T-cannula in the distal ileum. A cornstarch-based diet containing full-fat almonds as the sole AA source was used. The DE and ME of full-fat almonds were 4370 and 4320 kcal/kg (standard error = 276 and 300). The standardized ileal digestibility values of Lys, Met, Thr, and Trp in full-fat almonds were 95.2%, 61.6%, 69.4%, and 101.3% (standard error = 9.2 to 10.7). The standardized total tract digestibility of P in full-fat almonds was estimated to be 69.3% (standard error = 4.5). When full-fat almonds (0.400 USD/kg) were included at 5% in pig diets, feed cost decreased by 0.005 USD/kg. In conclusion, an Excel spreadsheet based on measured energy and nutrient concentrations in full-fat almonds enables the estimation of feed cost changes according to ingredient prices and full-fat almond inclusion rates.

## Simple Summary

Full-fat almonds are considered an alternative feedstuff for pigs due to their large amounts of energy and nutrients. The purposes of the present work were to measure the energy values and digestibility of amino acids and phosphorus in full-fat almonds and to assess their potential to replace conventional ingredients in pig diets based on corn and soybean meal. In animal experiments, the energy utilization and nutrient digestibility of full-fat almonds were determined using growing pigs. The metabolizable energy content was 4320 kcal/kg, and the ileal digestibility values of Lys, Met, Thr, and Trp were 95.2%, 61.6%, 69.4%, and 101.3%, respectively. The digestibility of phosphorus was 69.3%. Based on the energy and nutrient concentrations in full-fat almonds, a model was developed to calculate feed cost changes when full-fat almonds replace conventional ingredients. The inclusion of full-fat almonds (0.400 USD/kg) at 5% reduced feed cost by approximately 0.005 USD/kg. The model can estimate the economic value of full-fat almonds for pigs at different prices and inclusion rates.

## 1. Introduction

Feed cost accounts for the largest proportion of total swine production costs. For economical diet formulation, an accurate nutritional evaluation for feed ingredients is critical [1]. In particular, the dietary concentrations of energy, amino acids (AA), and phosphorus (P) are the most important considerations in swine feed formulation. Before incorporating an alternative ingredient into swine diets, the energy and nutrient utilization in the alternative ingredient should be evaluated for its nutritional suitability, cost-effectiveness, and market availability [2].

Full-fat almonds are considered an alternative ingredient because of their high fat and energy concentrations [3]. Although almonds are primarily used for human consumption [4], almonds failing to meet human-grade standards or unsuitable for commercial products despite being safe for consumption can be available for use in animal diets. A previous experiment reported that replacing animal fat with full-fat almonds in diets increased growth performance and nutrient digestibility in broilers [5]. However, to our knowledge, digestible energy (DE), metabolizable energy (ME), and the digestibility of AA and P in full-fat almonds have not been documented for pigs.

To determine the economic values of an ingredient, the replacement coefficients (RC) can be used for defining the proportion of conventional feed ingredients replaced by the alternative feedstuff without changes in the overall nutrient concentrations of the diet [2,6]. The calculation of RC is necessary for determining the values of an alternative feed ingredient. The value of cashew nuts as an alternative feedstuff was determined for pigs using the RC in a previous study [2]. However, to our knowledge, the values of full-fat almonds in pig diets have not been reported. Therefore, the objectives of the present experiment were to determine the DE and ME, the standardized ileal digestibility (SID) of AA, and the standardized total tract digestibility (STTD) of P in full-fat almonds fed to growing pigs and to calculate the replacement value of full-fat almonds in diets based on corn and soybean meal (SBM).

## 2. Materials and Methods

The procedures for the animal experiment were approved by the Institutional Animal Care and Use Committee of Konkuk University (Seoul, Republic of Korea, KU24257 and KU25054).

### 2.1. Determination of the Utilization of Energy and Nutrients in Full-Fat Almonds for Pigs

#### 2.1.1. Animals, Diets, and Experimental Design

Full-fat almonds sourced from Easy Bio System Inc. (Seoul, Republic of Korea) contained 5616 kcal/kg gross energy (GE), 17.1% crude protein (CP), 30.7% ether extract (EE), and 32.4% amylase-treated neutral detergent fiber (Table 1). In experiment 1, six crossbred barrows (Landrace × Yorkshire) with an initial body weight (BW) of 34.5 kg (standard deviation = 2.0) were used to determine the DE and ME in full-fat almonds and the apparent total tract digestibility (ATTD) of GE and nutrients in the experimental diets. The pigs were individually housed in metabolism crates equipped with a feeder, a fully slatted floor, and a urine tray allowing the complete and separate collection of feces and urine from each pig. A 2-period crossover design was used with 6 animals, 2 experimental diets, and 2 periods to obtain 6 observations for each experimental diet. The experimental diets were a corn–SBM-based diet and a diet containing 10% full-fat almonds (Table 2). In the preliminary palatability test using cornstarch-based diets containing 5%, 10%, or 20% full-fat almonds, with 3 barrows of 17-kg BW and 3 periods to obtain 3 observations for each diet, the mean feed refusal rates were 0%, 10.2%, and 36.7% for diets containing 5%, 10%, and 20% full-fat almonds, respectively. Therefore, the inclusion rate of full-fat almonds in the experimental diet was set at 10%, which was considered unlikely to substantially affect the feed intake of pigs. The energy sources in the basal diet were corn, SBM, and soybean oil, and their ratio was kept constant in both experimental diets. Vitamins and minerals were included in all diets to meet or exceed the nutrient requirement estimates recommended by the NRC [7].

In experiment 2, six crossbred barrows (Landrace × Yorkshire) with an initial BW of 28.9 kg (standard deviation = 1.0) were used to determine the SID of AA and STTD of P in full-fat almonds. The pigs were individually housed in pens (1.2 m × 1.6 m) equipped with a feeder and a nipple drinker. The experimental diet based mainly on cornstarch and sucrose contained 10% full-fat almonds as the sole source of AA in the diet (Table 2). Six observations were obtained. Chromium oxide was included in the diet at 0.5% as an indigestible index. In both experiments, all experimental diets were prepared using a flat-bottom mixer (DKM-450SU; Daegwang Construction Mechanic Inc., Seoul, Republic of Korea), with 42 revolutions per minute and a 450-L capacity, and they were provided to pigs in a mash form.

#### 2.1.2. Feeding and Sample Collection

In both experiments, pigs were fed the experimental diet at the quantity of 3.5% BW per day. The daily feed allowance was divided into two equal meals and provided at 0800 and 1700 h. Water was always accessible. The feed allowance for each pig was adjusted according to the BW at the beginning of each period.

In experiment 1, each experimental period consisted of a 5-d adaptation period and a 4-d collection period. To enable quantitative fecal collection, the marker-to-marker procedure was employed [8]. Chromic oxide was used as a marker for the initiation and termination of fecal collection. The marker was mixed with the morning meal at 0.5% on d 6 and d 10 of each experimental period. Fecal collection was initiated when the marker color was observed in the feces and terminated when the color appeared again in the feces. All collected feces were immediately stored in a freezer at −20 °C. From 1400 h on d 6 to 1400 h on d 10, urine was continuously collected in a bucket with 200 mL of 6 *N* HCl to inhibit nitrogen volatilization [9]. The collected feces were dried in a drying oven at 55 °C and finely ground to pass through a 1 mm screen.

In experiment 2, during the experimental period, a 4-d adaptation period was followed by a 3 d-ileal digesta collection period. The ileal digesta were collected from 0830 to 1830 h on the collection days in a Whirl-Pak bag (NASCO, Fort Atkinson, WI, USA) attached to the T-cannula. The bags were replaced every 30 min or whenever they became full. To minimize fermentation of AA, the collected ileal digesta were immediately stored in a freezer at −20 °C. Before the chemical analyses, the ileal digesta samples were lyophilized and ground to pass through a 1-mm screen.

#### 2.1.3. Chemical Analyses

According to the AOAC [10], dry matter (DM; method 930.15), ash (method 942.05), and CP (method 990.03) concentrations in the ingredients (corn, SBM, and full-fat almonds), experimental diets, feces, and ileal digesta were measured. The ether extract (method 920.39), amylase-treated neutral detergent fiber (method 2002.04), and acid detergent fiber (method 973.18) in the ingredients and experimental diets were determined [10]. The GE contents in the ingredients, experimental diets, feces, and urine were measured using bomb calorimetry (Parr 6400; Parr Instruments Co., Moline, IL, USA). For the urine GE determination, urine samples were dried according to a method suggested by Son et al. [11]. Briefly, frozen urine was thawed, and approximately 3 mL was added to a 0.3–0.4 g cotton ball in a stainless-steel crucible. Then, the urine in the crucible was lyophilized for 24 h, and urinary GE was determined using bomb calorimetry. Chromium concentrations in the almond diet and ileal digesta were measured (method 990.08) using UV/Vis spectrophotometry (Optizen 2120UV; Mecasys Inc., Daejeon, Republic of Korea) according to the AOAC [10]. The samples were digested by the wet digestion method in the presence of perchloric acid (method 957.02), and the concentrations of calcium in full-fat almonds and almond diet and P in full-fat almonds, almond diet, and ileal digesta were determined by using an inductively coupled plasma spectroscopy (method 985.01) according to the AOAC [10]. The AA concentrations in full-fat almonds and ileal digesta were also determined by hydrolyzing with 6 *N* HCl for 24 h at 110 °C (method 994.12) according to the AOAC [10]. Methionine and Cys were measured as Met sulfone and cysteic acid following cold performic acid oxidation overnight [method 982.30 E(b)]. Tryptophan was analyzed after hydrolysis with NaOH for 22 h at 110 °C [method 982.30 E(c)].

#### 2.1.4. Calculations and Statistical Analyses

In experiment 1, the DE and ME concentrations and the ATTD of DM, organic matter, and CP in the experimental diets were calculated as described by Kim et al. [12]. The DE and ME of full-fat almonds were calculated using the difference procedure [8].

In experiment 2, the apparent ileal digestibility (AID) values of CP, AA, and P in full-fat almonds were calculated using the chromium concentrations in the almond diet and ileal digesta as described by Oh et al. [13]. The SID of CP and AA in full-fat almonds were determined by correcting the AID with the basal endogenous losses of CP and AA (g/kg of DM intake) estimated using prediction equations developed by Park et al. [14]. The STTD of P in the almond diet was calculated by accounting for basal ileal endogenous P losses [15] and hindgut P disappearance [16]. Subsequently, the STTD of P in full-fat almonds was calculated using the STTD value of P in dicalcium phosphate (DCP) reported by Lee et al. [17] and the difference procedure [8]. The standard error (SE) of each mean value was calculated for the data of energy and nutrient utilization in full-fat almonds.

Data from experiment 1 were statistically analyzed using the GLM procedure of SAS 9.4 (SAS Inst. Inc., Cary, NC, USA). The statistical model included diet. Outliers were defined as observations deviating by more than 1.5 times the interquartile range from the 1st or 3rd quartiles, and no outliers were found. Least-squares means were calculated for each dietary treatment. The experimental unit was an individual pig, and statistical significance was declared at *p* < 0.05.

### 2.2. Determination of the Replacement Value and Economic Analysis of Full-Fat Almonds

#### 2.2.1. Using Full-Fat Almonds as an Alternative Feed Ingredient

The potential of full-fat almonds as an alternative to conventional feed ingredients, such as corn and SBM, and fat sources, such as choice white grease, was evaluated. The energy and nutrient compositions of corn, SBM, and choice white grease were obtained from the NRC [7], whereas the energy and nutrient composition of full-fat almonds was based on the values determined in the present experiments.

#### 2.2.2. Calculation of the Economic Value of Full-Fat Almonds

To determine the economic value of an alternative feedstuff, the price difference was compared when an ingredient replaces conventional feedstuffs, maintaining the nutrient concentrations of a diet [2]. As the nutrient composition of an alternative feedstuff is different from that of conventional feedstuffs, balancing the ME and nutrient composition by adjusting the inclusion rates of other ingredients is needed to meet the nutritional requirements of pigs when an alternative feed ingredient is incorporated into a pig diet. Consequently, corn, SBM, choice white grease, _L_-Lys·HCl, _DL_-Met, _L_-Thr, _L_-Trp, limestone, and DCP were used, along with full-fat almonds, to maintain the ME and nutrient composition of the diet in the present work.

#### 2.2.3. Spreadsheet Configuration

To enable the calculation of the economic value of full-fat almonds, 3 worksheets in Microsoft^®^ Excel 2020 were developed. In worksheet 1, the nutritional profiles of the feedstuffs were provided. Then, worksheet 2 was developed to balance ME and nutrient concentrations using corn, SBM, and other ingredients while setting full-fat almonds as 100%. This enabled the determination of the RC of conventional feedstuffs for full-fat almonds. To balance nutrient concentrations when using full-fat almonds in pig diet formulations, the replacement value was calculated based on the ME, SID Lys, SID Met, SID Thr, SID Trp, total calcium, and STTD P. In worksheet 3, the feed price change (USD/kg) was calculated based on the inclusion rate of full-fat almonds and the price of the feed ingredients.

## 3. Results

### 3.1. Utilization of Energy and Nutrients in Full-Fat Almonds for Growing Pigs

In both experiments, all pigs remained healthy and readily consumed their diets. The ATTD of GE in the almond diet was less (*p* = 0.043) than that in the basal diet, whereas the DE and ME concentrations in the almond diet were greater (*p* < 0.05) than those in the basal diet (Table 3).

The DE and ME concentrations in full-fat almonds for pigs were 4370 and 4320 kcal/kg on an as-fed basis (SE = 276 and 300), respectively (Table 4). The DE:GE, ME:GE, and ME:DE in full-fat almonds for pigs were 0.778, 0.769, and 0.987 (SE = 0.049, 0.053, and 0.008), respectively.

The ATTD of DM and organic matter in the almond diet fed to pigs was less (*p* < 0.05) than that in the basal diet (Table 5). No difference was observed in the ATTD of CP between the basal diet and almond diet fed to pigs.

The SID of CP in full-fat almonds for pigs was 64.2% (SE = 9.3), and the SID values of indispensable AA in full-fat almonds for pigs were 95.2% for Lys (SE = 10.7), 61.6% for Met (SE = 10.0), 69.4% for Thr (SE = 9.2), and 101.3% for Trp (SE = 9.2; Table 6). The STTD of P in full-fat almonds for pigs was 69.3% (SE = 4.5).

### 3.2. Replacement Coefficient of Full-Fat Almonds in Swine Diets

The concentrations of ME, SID AA, total calcium, and STTD P in full-fat almonds measured in experiments 1 and 2 were entered into worksheet 1 (Figure 1; Supplementary File S1). Based on the values in worksheet 1, worksheet 2 consisted of two tables, upper and lower, for calculating the RC for full-fat almonds (Figure 2; Supplementary File S1).

In worksheet 2, the upper part of the spreadsheet included the replacement values based on the energy and nutrient compositions of full-fat almonds and crystalline AA, whereas the lower part of the spreadsheet included those of corn, SBM, choice white grease, limestone, and DCP to be replaced with full-fat almonds and AA. The replacement values for feed ingredients and supplements were adjusted to balance energy and nutrient concentrations, while the replacement value of full-fat almonds was kept at 100%. The replacement values obtained in this spreadsheet were used as RC in the following equation:

1 × full-fat almonds + 0.00886 × _L_-Lys·HCl + 0.00229 × _DL_-Met + 0.00378 × _L_-Thr + 0.00151 × _L_-Trp  = 0.41986 × corn + 0.37933 × SBM + 0.21165 × choice white grease + 0.00155 × limestone + 0.00405 × DCP

where full-fat almonds with an RC of 1 supplemented with _L_-Lys·HCl at 0.00886, _DL_-Met at 0.00229, _L_-Thr at 0.00378, and _L_-Trp at 0.00151 are equivalent to corn at 0.41986, SBM at 0.37933, choice white grease at 0.21165, limestone at 0.00155, and DCP at 0.00405 to maintain the same ME and nutrient concentrations. In worksheet 3, the prices of full-fat almonds, corn, SBM, and other ingredients were input into the table based on recent market prices in USD as examples (Figure 3, Figure 4 and Figure 5; Supplementary File S1).

In the Excel file, a user can update the prices of the ingredients. The contribution of each ingredient to the diet is based on the inclusion rate of full-fat almonds. The increased price due to the inclusion of full-fat almonds and crystalline AA is calculated, and the decreased price by reducing the inclusion rates of corn, SBM and other ingredients is also calculated. The cost saving by including full-fat almonds in pig diets while maintaining ME and nutrient concentrations is also calculated using the following equation:

Cost saving (USD/kg) = full-fat almonds inclusion rate (%) × [0.41986 × corn price + 0.37933 × SBM price + 0.21165 × choice white grease price + 0.00155 × limestone price + 0.00405 × DCP price − (1 × full-fat almonds price + 0.00886 × _L_-Lys·HCl price + 0.00229 × _DL_-Met price + 0.00378 × _L_-Thr price + 0.00151 × _L_-Trp price)]/100%

where the unit for the price of each ingredient is USD/kg.

With an inclusion rate of 10% and a market price of 0.400 USD/kg for full-fat almonds, the feed cost decreased by approximately 0.009 USD/kg based on the present model (Figure 3). By reducing the inclusion rate of full-fat almonds to 5% of diets, the cost decreased by approximately 0.005 USD/kg (Figure 4). The market price of full-fat almonds that resulted in no change in feed costs when formulating pig diets was 0.500 USD/kg (Figure 5).

## 4. Discussion

Almonds are widely consumed around the world, either directly or as an ingredient in a variety of food products. Approximately 80% of the world’s almonds are produced in the United States, with California being the primary production region. In California alone, almond production exceeded 1.1 billion kg annually between 2021 and 2025, reaching approximately 1.36 billion kg in 2025 [4]. Because almonds contain a high fat content with large quantities of oleic acid and linoleic acid [18], almonds potentially have merit as an alternative feed ingredient in animal diets. Although the ATTD of GE and nutrients in pigs fed almond meal derived from nut residues and broken almonds was previously reported [19], information on the energy and nutrient digestibility of full-fat almonds in pigs remains limited. Therefore, the present work aimed to determine the energy and nutrient utilization of full-fat almonds in growing pigs and to calculate the replacement value of full-fat almonds in pig diets for the evaluation of their economic value.

The GE concentration in full-fat almonds used in the present experiment was within the range reported in previous studies [18,19,20], which varied across the studies, likely due to differences in EE concentrations. The EE concentration in full-fat almonds varies depending on the almond variety and whether the shell or skin is present [18]. The variable EE concentration of full-fat almonds should be considered when using this ingredient in diet formulations because EE concentration is highly correlated with the energy value. Moreover, the high EE content of full-fat almonds may adversely affect feed manufacturing by reducing feed flowability [2]. The concentrations of ash, calcium, and P in full-fat almonds used in the present experiment were comparable to the values reported in previous experiments [5,18], whereas the AA concentrations and the Lys-to-CP ratio in full-fat almonds were less than the values in previous reports [18,21]. In addition, although Yada et al. [22] reported that the total dietary fiber concentrations in almond varieties produced in California ranged from 7.9% to 16.0% on an as-is basis, the full-fat almonds used in the present experiment contained relatively high fiber concentrations, which may be attributed to the substantial inclusion of shell and skin in the ingredient. These differences in nutrient composition among full-fat almonds may be attributed to genetic and ecological factors, as well as processing conditions, including shell removal, skin peeling, and roasting [4].

The low ATTD of GE, DM, and organic matter in the almond diet compared with the corn–SBM-based diet observed in experiment 1 may be attributed to its relatively high fiber content (11.8% vs. 10.0% amylase-treated neutral detergent fiber). Previous experiments reported that energy and nutrient digestibility in pig diets can decrease with increasing dietary fiber content [11,23]. In contrast, the greater DE in the almond diet observed in the present experiment was most likely because the almond diet contained a greater GE concentration than the corn–SBM-based diet (4131 vs. 3933 kcal/kg). Additionally, the greater ME in the almond diet can be explained by the greater DE in the almond diet, together with similar CP contents between the basal and almond diets, resulting in no differences in urinary GE losses [11].

The DE and ME concentrations of full-fat almonds determined in the present experiment were greater than the NRC-reported DE (3451 kcal/kg) and ME (3395 kcal/kg) values of corn [7], a major energy source in swine diets, indicating that full-fat almonds may partially replace conventional energy sources in pig diets. However, when dietary inclusion rates of 5%, 10%, and 20% were preliminarily tested to determine an appropriate inclusion rate of full-fat almonds in the experimental diets, feed refusal was observed in one pig at 10%, and the feed intake of pigs fed the 20% almond diet decreased compared with those fed at 5% or 10% inclusion of full-fat almonds. Therefore, feed intake should be carefully considered when incorporating full-fat almonds into the diets [5]. In addition, the SID values of Lys, Trp, and Val in full-fat almonds fed to pigs were greater than 95%, which were greater than the NRC-reported SID values of Lys (89%), Trp (91%), and Val (87%) in SBM [7], a major protein source used in swine diets. Although the SID AA concentrations of full-fat almonds are less than those of SBM, the dietary inclusion of full-fat almonds may also provide a partial contribution of digestible AA. Because the concentrations of available energy, AA, and P in full-fat almonds determined in the present experiment were measured using approximately 30-kg growing pigs, the BW of pigs should also be considered when incorporating full-fat almonds into diets, as the utilization of energy and nutrients can differ depending on the BW of pigs [24]. Further research is warranted to determine the optimal dietary inclusion rate of full-fat almonds for maintaining growth performance of pigs and to evaluate their nutrient utilization using pigs with different BW.

The SID values of Trp and Tyr in full-fat almonds measured in the present experiment were greater than 100% in experiment 2, which can be explained by the greater contribution of basal endogenous AA losses due to the low AA intake in pigs. Because full-fat almonds were included at 10% as the sole AA source in the cornstarch-based experimental diet, the amounts of Trp and Tyr were relatively low in the diet. Consistent with the observation in the present work, the previous study reported SID values for AA exceeding 100% when the test ingredient was included in the diet at only 15% [25]. In the case of Pro, the negative value of ileal Pro digestibility in full-fat almonds for pigs was observed in the present experiment, which is consistent with the negative value in bakery byproducts reported by Oh et al. [13]. In contrast, Stein et al. [26] reported SID values for Pro in corn and SBM for pigs that exceeded 100%. This discrepancy among studies can be partially explained by the variability in basal endogenous Pro losses in pigs. Stein et al. [26] measured endogenous Pro losses using an N-free diet, which can overestimate endogenous losses of Pro. Conversely, in the present experiment and Oh et al. [13], the previously developed model for estimating basal endogenous Pro losses was used to calculate the SID of Pro in feed ingredients for pigs [14]. The very low values of ileal Pro digestibility observed in the present work using the prediction model may be attributed to endogenous Pro losses derived from sloughed epithelial cells [13,26]. Additionally, fibrous ingredients in the pig diet can increase ingredient-specific endogenous losses, including AA-rich sloughed epithelial cells, mucin, and digestive secretions, and Pro-rich mucin is highly resistant to proteolysis in the intestine, which can further contribute to the substantial amounts of Pro losses [27]. Moreover, the dietary inclusion rate of full-fat almonds used in the present experiment was relatively low at 10%, which may have partially contributed to the variability in the measured value [12,25].

The RC model developed in the present experiment provides a simplified tool for assessing the applicability of alternative feed ingredients in swine diets. The model balances SID AA concentrations using crystalline AA and also accounts for ME, total Ca, and STTD P to meet the nutritional requirements of pigs [2,6]. The market price and nutritional composition of alternative ingredients, including full-fat almonds, may vary depending on supply chain logistics, regional availability, market demand, growing conditions, and processing methods [3,18,20]. Therefore, these variations should be considered when formulating swine diets. The Excel-based program allows users to readily update ingredient nutritional values and prices, facilitating flexible evaluation of nutrient balance and feed cost in different formulation scenarios. No studies have reported an optimal dietary inclusion rate of full-fat almonds for pigs; however, the NRC [7] suggested that the maximum inclusion of added fat in pig diets is approximately 6% because of feed-handling characteristics under practical conditions. Therefore, the present experiment used the inclusion rate of 5% for full-fat almonds in the replacement value calculator, considering their relatively low palatability, high fiber content, and variability in the EE concentration of almond ingredients [18]. Furthermore, from an economic perspective, full-fat almonds were considered cost-effective when their market price was below 0.500 USD/kg in the present work. However, not all digestible indispensable AA, CP, fiber, or other nutrients in feed ingredients were considered when determining the replacement value in the present experiment. In addition, increasing the inclusion of full-fat almonds in swine diets may affect nutrient digestibility, ingredient interactions, and growth performance for pigs, which were not accounted for in the present model. Furthermore, because growth performance was not evaluated in the present experiments, the 5% inclusion rate may not be interpreted as an optimal dietary inclusion rate of full-fat almonds. Because variations in processing methods and regional practices also influence the nutritional composition of full-fat almonds and their nutritional and economic value in pig diets [4,18], further research is warranted to determine the potential interaction between full-fat almonds and other ingredients when fed to pigs and to evaluate the potential use of other almond-derived products in pig diets.

## 5. Conclusions

The present experiment determined the energy and nutrient utilization of full-fat almonds as a novel alternative feed ingredient in swine diets. The Excel spreadsheet developed using the energy and nutrient concentrations of full-fat almonds measured in the present work enables the estimation of changes in feed cost by updating the ingredient prices and the inclusion rate of full-fat almonds. When the market price of full-fat almonds is 0.400 USD/kg, a 5% inclusion rate in swine diets results in feed cost reduction of approximately 0.005 USD/kg. Full-fat almonds can be an alternative feed ingredient for pigs, offering both nutritional and economic benefits when included at appropriate rates. Further research is needed to determine the optimal inclusion rate of full-fat almonds in swine diets, considering variation in the nutrient composition of full-fat almonds, interactions with other feed ingredients, and the growth performance of pigs.

## Figures and Tables

**Figure 1:**
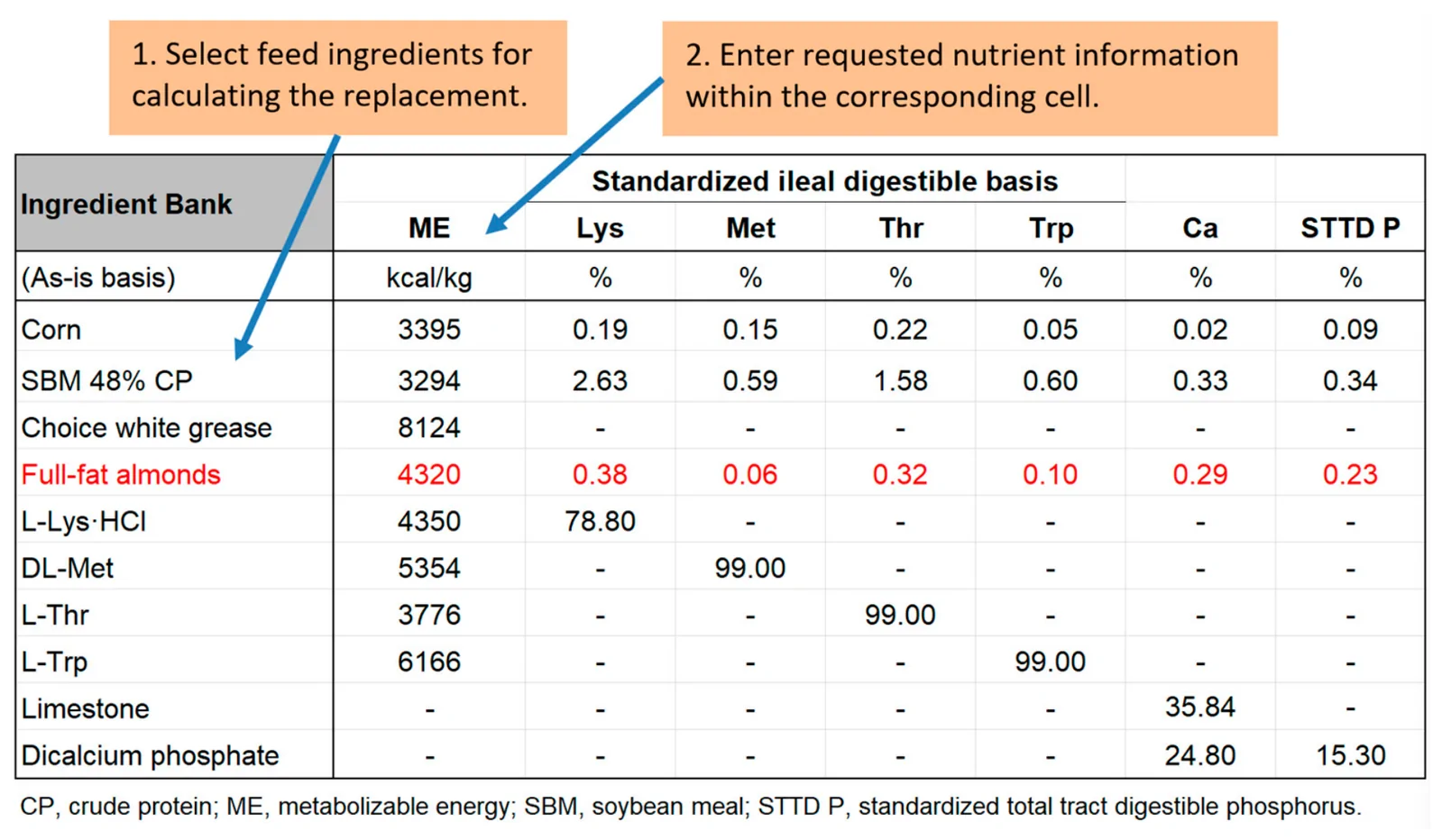
Nutritional composition of full-fat almonds and other feed ingredients used in the present experiment (as-is basis).

**Figure 2:**
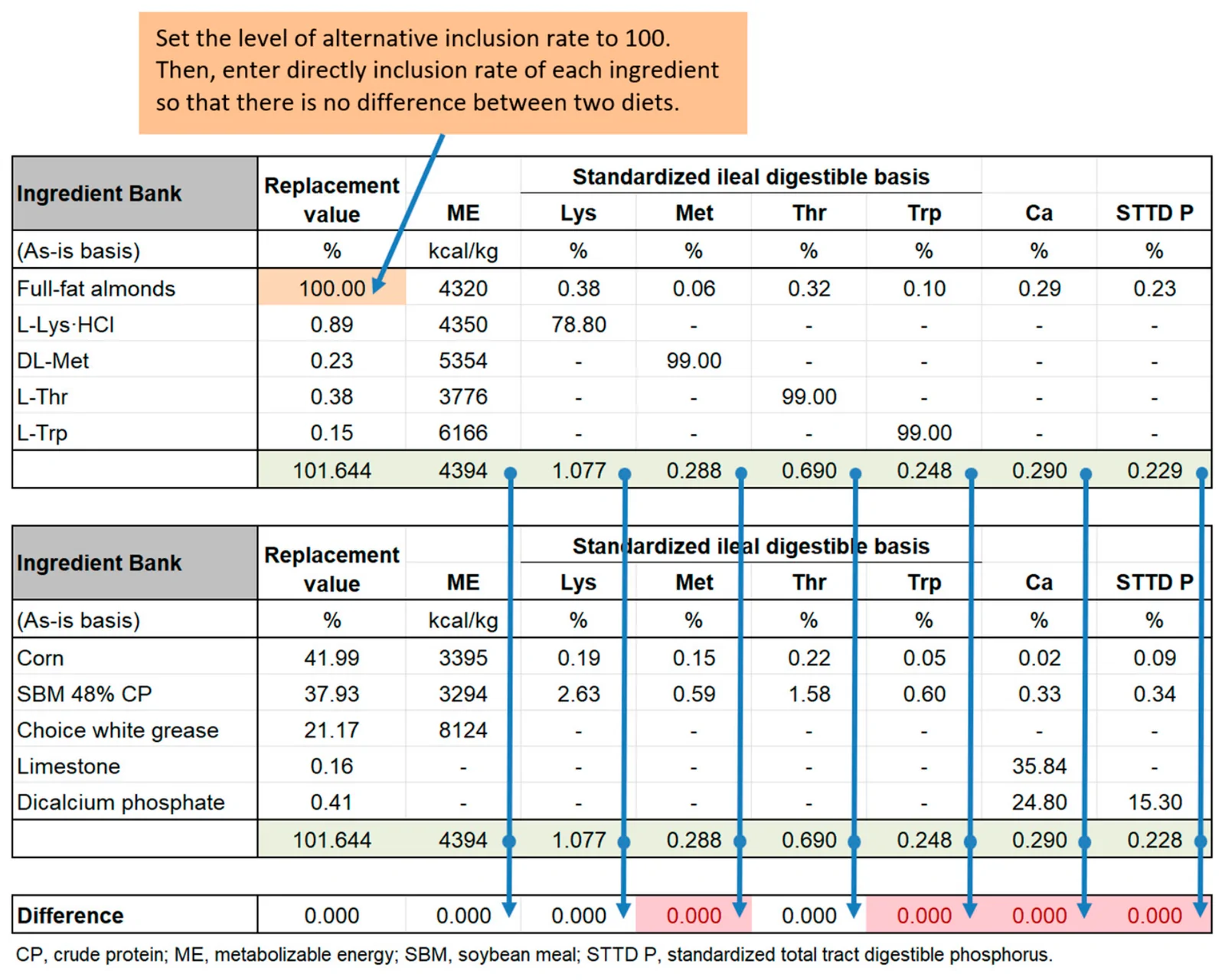
The calculation of the replacement value of full-fat almonds. The inclusion rates of the ingredients were adjusted to maintain the energy and nutrient concentrations.

**Figure 3:**
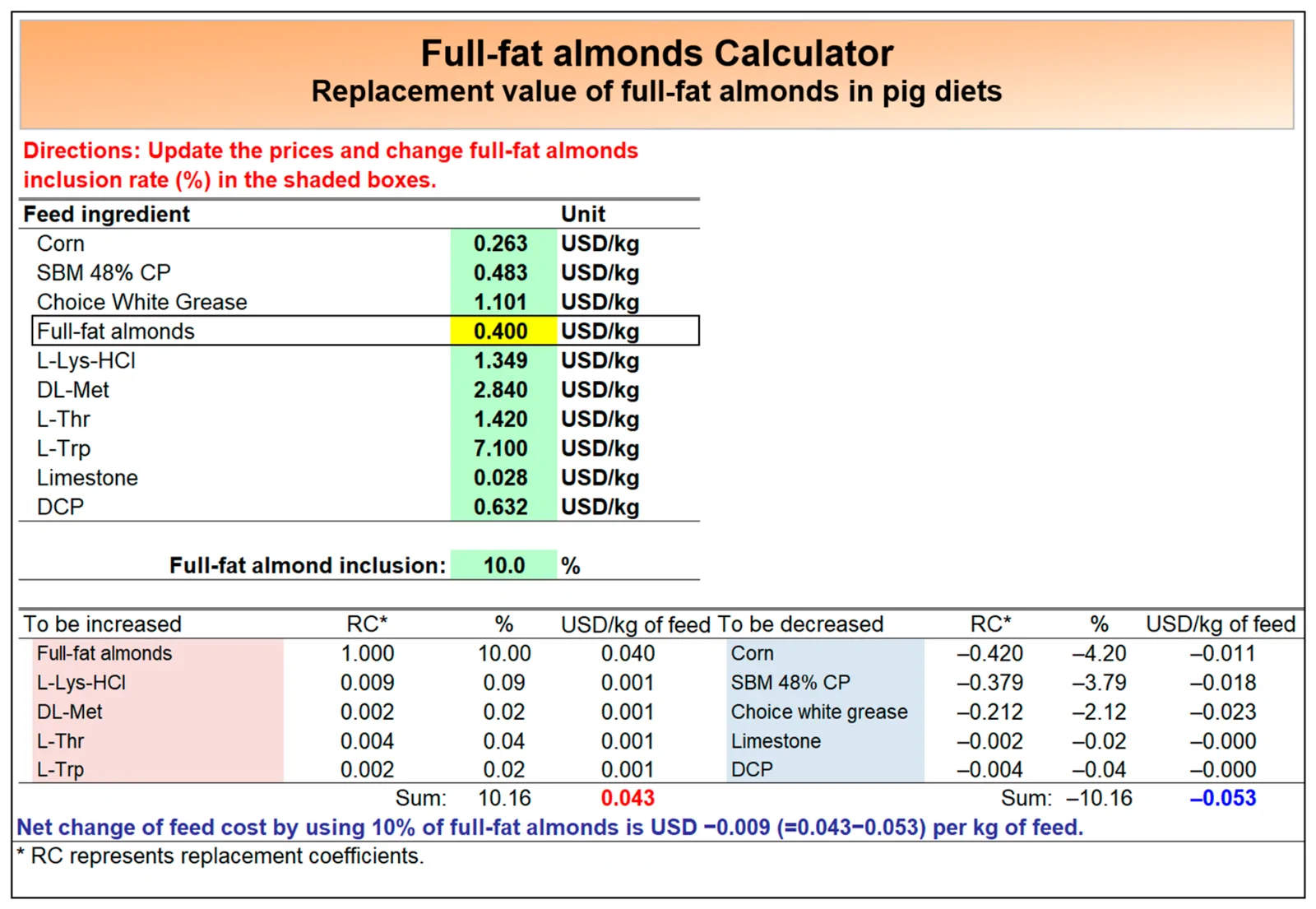
A sample calculation of the difference in feed price when the price of full-fat almonds was 0.400 USD/kg at the inclusion rate of 10%. The net change in feed price by using 10% full-fat almonds was −0.009 USD/kg (=0.043–0.053).

**Figure 4:**
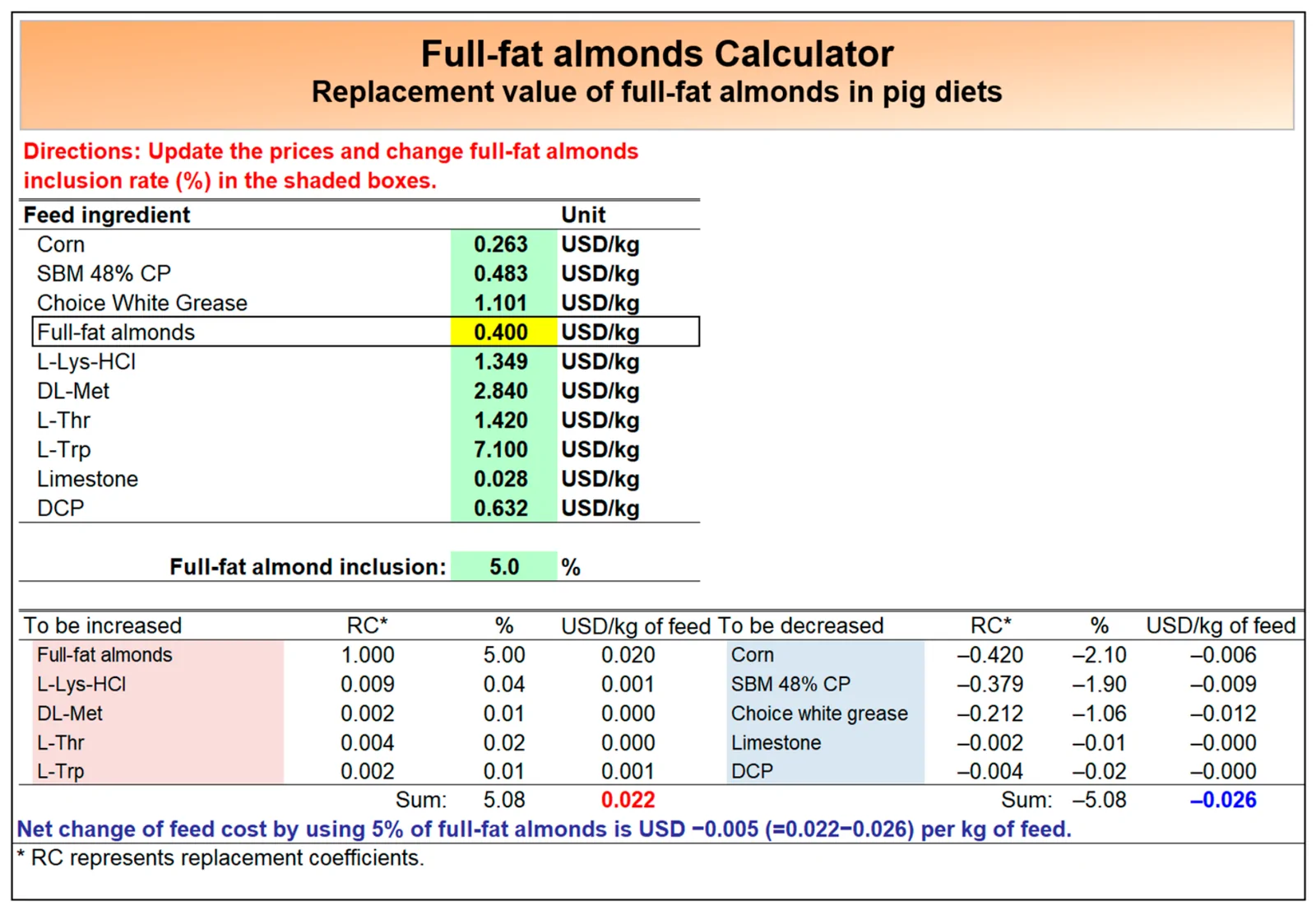
Feed cost calculations when the price of full-fat almonds was 0.400 USD/kg at the inclusion rate of 5%. The net change in feed price by using 5% full-fat almonds was −0.005 USD/kg (=0.022–0.026).

**Figure 5:**
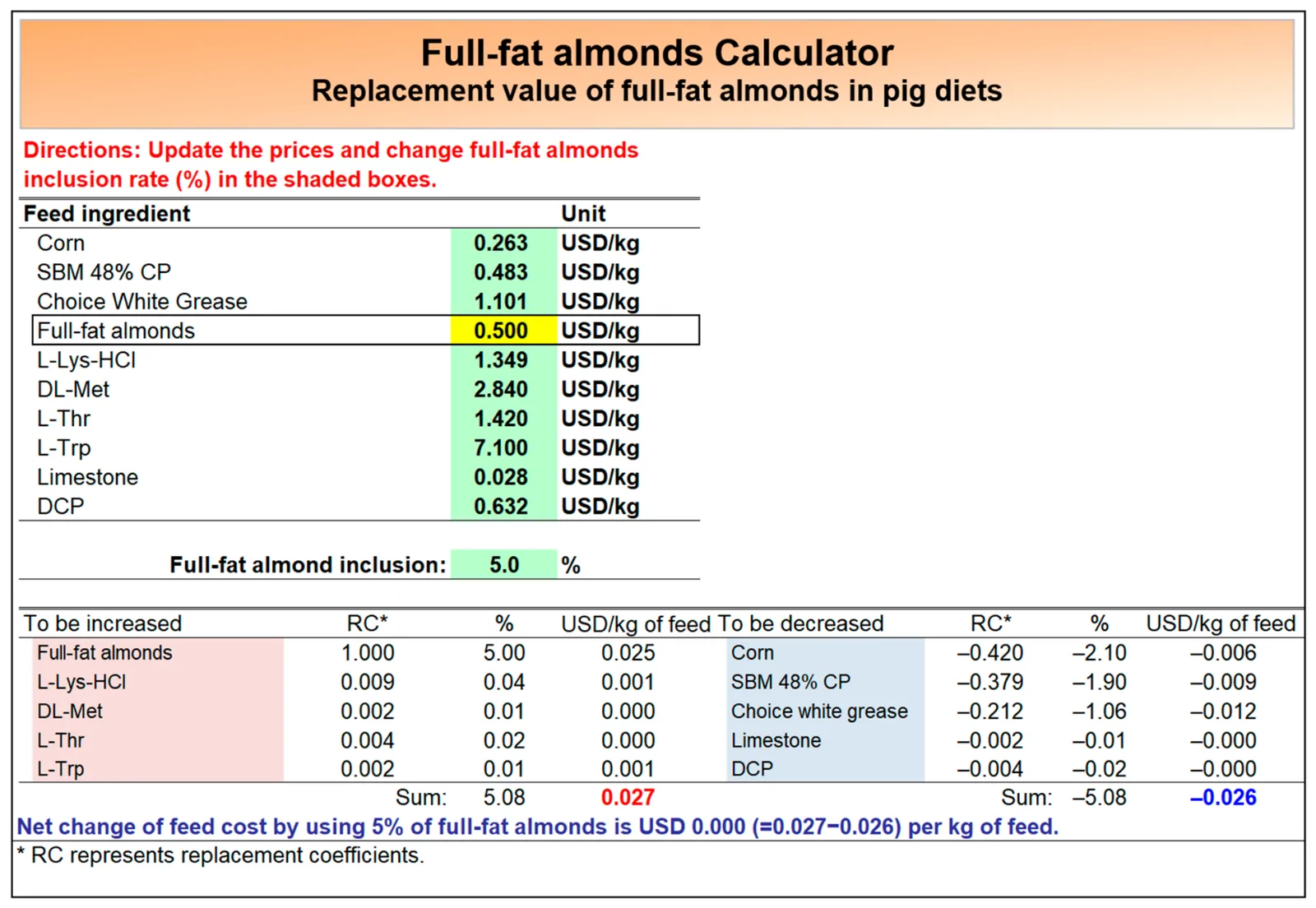
Feed cost calculations when the price of full-fat almonds was 0.500 USD/kg at the inclusion rate of 5%. The net change in feed price by using 5% full-fat almonds was 0 USD/kg (=0.027–0.026). This suggests that the price of full-fat almonds needs to be less than 0.500 USD/kg for cost-effectiveness.

**Table 1:** Analyzed chemical compositions of corn, soybean meal, and full-fat almonds (as-is basis).

Item, %	Corn	Soybean Meal	Full-Fat Almonds
Dry matter	89.04	91.00	91.84
Gross energy, kcal/kg	3886	4222	5616
Crude protein	8.34	42.53	17.08
Ether extract	2.42	1.22	30.65
Ash	1.48	6.59	3.73
Phosphorus	- ^1^	-	0.33
Calcium	-	-	0.29
Amylase-treated neutral detergent fiber	8.67	13.37	32.40
Acid detergent fiber	3.29	7.29	21.54
Indispensable amino acids			
Arginine	-	-	1.19
Histidine	-	-	0.35
Isoleucine	-	-	0.44
Leucine	-	-	0.93
Lysine	-	-	0.40
Methionine	-	-	0.10
Phenylalanine	-	-	0.61
Threonine	-	-	0.46
Tryptophan	-	-	0.10
Valine	-	-	0.45
Dispensable amino acids			
Alanine	-	-	0.65
Aspartic acid	-	-	1.46
Cysteine	-	-	0.15
Glutamic acid	-	-	3.17
Glycine	-	-	0.81
Proline	-	-	0.49
Serine	-	-	0.62
Tyrosine	-	-	0.40

^1^ Not determined.

**Table 2:** Ingredients and chemical compositions of experimental diets (as-fed basis; experiments 1 and 2).

Item	Experiment 1		Experiment 2
	Basal Diet	Almond Diet	Almond Diet
Ingredient composition, %			
Ground corn	76.9	69.0	-
Soybean meal, 43% crude protein	18.5	16.6	-
Soybean oil	2.0	1.8	-
Full-fat almonds	-	10.0	10.0
Cornstarch	-	-	66.6
Sucrose	-	-	20.0
Ground limestone	0.8	0.8	0.3
Dicalcium phosphate	1.2	1.2	2.0
Sodium chloride	0.3	0.3	0.3
Vitamin–mineral premix ^1^	0.3	0.3	0.3
Chromic oxide	-	-	0.5
Analyzed chemical composition, %			
Dry matter	89.05	89.57	90.89
Gross energy, kcal/kg	3933	4131	4310
Crude protein	14.01	15.09	1.85
Ether extract	3.95	6.55	0.21
Ash	4.66	4.69	3.18
Phosphorus	- ^2^	-	0.41
Calcium	-	-	0.69
Amylase-treated neutral detergent fiber	9.98	11.79	3.50
Acid detergent fiber	3.49	5.87	2.10

^1^ Provided the following quantities per kilogram of complete diet: vitamin A as retinyl acetate, 18,000 IU; vitamin D_3_ as cholecalciferol, 3600 IU; vitamin E as _DL_-α-tocopheryl acetate, 60 IU; vitamin K as menadione nicotinamide bisulfite, 4.5 mg; thiamin as thiamin mononitrate, 4.5 mg; riboflavin, 7.5 mg; pyridoxine as pyridoxine hydrochloride, 4.5 mg; vitamin B_12_, 0.06 mg; _D_-pantothenic acid as _D_-calcium pantothenate, 30 mg; folic acid, 1.5 mg; niacin as nicotinamide, 45 mg; biotin, 0.3 mg; Co as cobaltous carbonate, 0.75 mg; Cu as copper sulfate, 60 mg; Fe as iron sulfate, 120 mg; I as calcium iodate, 0.68 mg; Mg as magnesium oxide, 60 mg; Mn as manganese sulfate, 60 mg; Se as sodium selenite, 0.3 mg; Zn as zinc sulfate, 60 mg. ^2^ Not determined.

**Table 3:** Energy utilization of experimental diets fed to growing pigs (as-is basis; experiment 1).

Item	Basal Diet	Almond Diet	SEM	*p*-Value
No. of observations	6	6	-	-
Feed intake, kg/d	1.31	1.31	0.05	0.988
Gross energy intake, kcal/d	5138	5392	220	0.434
Feces output, kg/d	0.168	0.195	0.010	0.097
Gross energy in feces, kcal/kg	4516	4592	24	0.052
Fecal gross energy output, kcal/d	761	895	48	0.075
ATTD of gross energy, %	85.2	83.4	0.6	0.043
Digestible energy in diet, kcal/kg	3351	3444	23	0.016
Urine output, kg/d	4.57	4.08	1.18	0.776
Gross energy in urine, kcal/kg	36	45	9	0.483
Urinary gross energy output, kcal/d	136	128	9	0.555
Metabolizable energy in diet, kcal/kg	3248	3347	25	0.021

ATTD = apparent total tract digestibility; SEM = standard error of the means.

**Table 4:** Energy values in full-fat almonds fed to growing pigs (experiment 1).

Item	Full-Fat Almonds	Standard Error
No. of observations	6	-
As-fed basis, kcal/kg		
GE	5616	-
DE	4370	276
ME	4320	300
Dry matter basis, kcal/kg		
GE	6115	-
DE	4758	301
ME	4704	326
Energy utilization		
DE:GE	0.778	0.049
ME:GE	0.769	0.053
ME:DE	0.987	0.008

GE = gross energy; DE = digestible energy; ME = metabolizable energy.

**Table 5:** Apparent total tract digestibility (ATTD) of nutrients in experimental diets fed to growing pigs (experiment 1).

Item	Basal Diet	Almond Diet	SEM	*p*-Value
No. of observations	6	6	-	-
ATTD of dry matter, %	86.2	84.1	0.5	0.021
ATTD of organic matter, %	87.7	85.5	0.5	0.015
ATTD of crude protein, %	81.8	82.0	0.7	0.823

SEM = standard error of the mean.

**Table 6:** Standardized ileal digestibility (SID) values of crude protein (CP) and amino acids (AA) and standardized total tract digestibility (STTD) values of phosphorus (P) in full-fat almonds fed to growing pigs (experiment 2).

Item	Full-Fat Almonds	Standard Error
No. of observations	6	-
SID of CP ^1^, %	64.2	9.3
SID of indispensable AA ^1^, %		
Arginine	69.3	4.7
Histidine	71.6	7.4
Isoleucine	61.0	6.5
Leucine	74.1	3.2
Lysine	95.2	10.7
Methionine	61.6	10.0
Phenylalanine	86.7	2.9
Threonine	69.4	9.2
Tryptophan	101.3	9.2
Valine	96.3	9.8
SID of dispensable AA ^1^, %		
Alanine	69.0	10.3
Aspartic acid	70.9	5.8
Cysteine	50.7	13.6
Glutamic acid	74.4	4.2
Glycine	53.7	14.5
Proline	−52.6	191.8
Serine	62.3	9.6
Tyrosine	151.5	2.8
STTD of P ^2^, %	69.3	4.5

^1^ Values for the SID values of CP and AA were calculated by correcting the apparent ileal digestibility values with basal endogenous losses of CP and AA using the models developed by Park et al. [14]. The estimated basal endogenous losses (g/kg dry matter intake) were: CP, 18.1; Arg, 0.65; His, 0.21; Ile, 0.34; Leu, 0.56; Lys, 0.46; Met, 0.10; Phe, 0.36; Thr, 0.58; Trp, 0.13; Val, 0.49; Ala, 0.64; Asp, 0.83; Cys, 0.21; Glu, 1.05; Gly, 1.69; Pro, 5.36; Ser, 0.61; Tyr, 0.59. ^2^ To estimate the STTD of P in full-fat almonds, the SID of P in the almond diet was first calculated by correcting the apparent ileal digestibility with basal ileal endogenous losses of P (177 mg/kg dry matter intake) [15]. The STTD of P in the diet was then estimated by correcting the SID of P with standardized hindgut P disappearance using the model developed by Park and Kim [16]. Finally, the STTD of P in full-fat almonds was calculated using the reported STTD value of P in dicalcium phosphate (87.0%) [17] and the difference procedure [8].

## Data Availability

The data are contained within this article or the Supplementary Materials.

## Supplementary Materials

The following supporting information can be downloaded at https://www.mdpi.com/article/10.3390/ani16193043/s1: Supplementary File S1: Full-fat Almonds Replacement Value Calculator_ver 1.2_Sep 14_2026.

## Abbreviations

The following abbreviations are used in this manuscript:

AA	Amino acid
AID	Apparent ileal digestibility
ATTD	Apparent total tract digestibility
BW	Body weight
CP	Crude protein
DCP	Dicalcium phosphate
DE	Digestible energy
DM	Dry matter
EE	Ether extract
GE	Gross energy
ME	Metabolizable energy
P	Phosphorus
RC	Replacement coefficient
SBM	Soybean meal
SE	Standard error
SID	Standardized ileal digestibility
STTD	Standardized total tract digestibility
